# Inflamatory dentigerous cyst – a clinical case

**DOI:** 10.1080/07853890.2021.1897349

**Published:** 2021-09-28

**Authors:** T. Nunes, A. Amaral, A. Mariz de Almeida, F. Salvado

**Affiliations:** a SAMS/SBSI – Serviço de Estomatologia, Lisboa, Portugal; b Clinica de Santa Madalena, Lisboa, Portugal; c Instituto Universitário Egas Moniz (IUEM), Egas Moniz Cooperativa de Ensino Superior, Caparica, Portugal; d Centro de Investigação Interdisciplinar Egas Moniz (CiiEM), Egas Moniz Cooperativa de Ensino Superior, Caparica, Portugal; e Universidade de Lisboa Faculdade de Medicina: Lisboa, Lisboa, Portugal

## Abstract

**Introduction:**

One of most frequent benevolent odontogenic cysts in the jaws is the dentigerous cyst. It usually occurs in the second and third decades of life and it´s barely seen in the childhood era [1]. They are unilocular and always radiolucent. They grow slowly and asymptomatic, unless there is a secondary infection, in this case they are associated with swelling and pain [2]. The dentigerous cyst of inflammatory origin is related with the inflammation present at the root apex of a non-vital primary tooth which spreads to involve the follicle of the unerupted immature permanent successor [3], as is in this case. Therapeutic modalities range from marsupialization and decompression to enucleation [2].

**Materials and methods:**

Male, 8 years old, appeared in the emergency appointment with pain and continuous abscess in the region of the second lower left deciduous molar. Extraoral examination revealed facial asymmetry. Intraoral examination revealed mixed dentition and in the lower left vestibule a hard, non-fluctuant swelling extending laterally from mesial surface of the mandibular first deciduous molar to the distal surface of the mandibular second deciduous molar, both with carious lesions and grade II mobility. Orthopantomography, revealed a well-circumscribed unilocular radiolucent lesion in the body of the jaw on the left side, which was associated with the crown of a vertically impacted second premolar with its subsequent displacement. Based on clinical and radiological findings, a provisional diagnosis of dentigerous cyst was made. We obtained the informed consent authorised by the ethics committee signed by the minor parents and let me reinforce that all the clinical case was made accordingly with the Helsinki Declaration of Ethical Principals. Firstly we did the extraction of the first and second lower left deciduous molar under local anaesthesia (2% Lidocaine with 1:1,000,000 Epinephrine), along with cyst enucleation through the extraction socket, preserving the impacted second premolar. The surgical piece was sent to histopathological examination in a tube with formaldehyde 37%.

**Results:**

Inflammatory dentigerous cysts was histopathological confirmed. Follow up period was 15 days, 1 month, 5 months and 10 months. After 15 days the patient referred substantial improvements on pain and the disappearing of the facial asymmetry as well as improvement on function, comfort and quality of life. It was then sent to Orthodontic treatment. At the 10 months follow up, it was removed the lingual arch due the eruption of definitive teeth. **Discussion and conclusions:** The dentigerous cysts are mostly found on routine radiographic examinations or by enlargement of affected region in the jaw with pain [3]. The pathogenesis of dentigerous cyst is still controversial but, one of the feasible proposed mechanisms is that the follicle of permanent successor might get secondarily infected from either periapical inflammation of a non-vital predecessor or other source leading to a dentigerous cyst formation [3]. Patient age, cyst size, site, involved dentition, and affected vital structures, are criteria which must be considered in the treatment modality, in this case we have a young patient and we opted for enucleation, without extraction of the impacted tooth [2]. This allow alleviation of cyst pressure to permit the retained teeth to erupt normally with root formation. Then the permanent tooth generally would erupt in the oral cavity naturally as this did. Sometimes orthodontic treatment is not needed. The patient should be followed until the complete eruption of permanent teeth in their designated location [3]. In the present clinic case the patience and indication for Ortodontic treatment for other reasons and now, presents the permanent teeth erupted.
